# Cardiac Sodium/Hydrogen Exchanger (NHE11) as a Novel Potential Target for SGLT2i in Heart Failure: A Preliminary Study

**DOI:** 10.3390/pharmaceutics14101996

**Published:** 2022-09-21

**Authors:** Lorena Pérez-Carrillo, Alana Aragón-Herrera, Isaac Giménez-Escamilla, Marta Delgado-Arija, María García-Manzanares, Laura Anido-Varela, Francisca Lago, Luis Martínez-Dolz, Manuel Portolés, Estefanía Tarazón, Esther Roselló-Lletí

**Affiliations:** 1 Clinical and Translational Research in Cardiology Unit, Health Research Institute Hospital La Fe (IIS La Fe), 46026 Valencia, Spain; 2 Cellular and Molecular Cardiology Research Unit, Department of Cardiology and Institute of Biomedical Research, University Clinical Hospital, 15706 Santiago de Compostela, Spain; 3 Cardiovascular Biomedical Research Center Network (CIBERCV), 28029 Madrid, Spain; 4 Department of Animal Medicine and Surgery, Veterinary Faculty, CEU Cardenal Herrera Unversity, 46115 Valencia, Spain

**Keywords:** SGLT2i, empagliflozin, heart failure, NHE1, NHE11, sodium channel

## Abstract

Despite the reduction of cardiovascular events, including the risk of death, associated with sodium/glucose cotransporter 2 inhibitors (SGLT2i), their basic action remains unclear. Sodium/hydrogen exchanger (NHE) has been proposed as the mechanism of action, but there are controversies related to its function and expression in heart failure (HF). We hypothesized that sodium transported-related molecules could be altered in HF and modulated through SGLT2i. Transcriptome alterations in genes involved in sodium transport in HF were investigated in human heart samples by RNA-sequencing. NHE11 and NHE1 protein levels were determined by ELISA; the effect of empagliflozin on NHE11 and NHE1 mRNA levels in rats’ left ventricular tissues was studied through RT-qPCR. We highlighted the overexpression of *SLC9C2* and *SCL9A1* sodium transport genes and the increase of the proteins that encode them (NHE11 and NHE1). NHE11 levels were correlated with left ventricular diameters, so we studied the effect of SGLT2i on its expression, observing that NHE11 mRNA levels were reduced in treated rats. We showed alterations in several sodium transports and reinforced the importance of these channels in HF progression. We described upregulation in NHE11 and NHE1, but only NHE11 correlated with human cardiac dysfunction, and its levels were reduced after treatment with empagliflozin. These results propose NHE11 as a potential target of SGLT2i in cardiac tissue.

## 1. Introduction

Heart failure (HF) continues to be a public health problem in industrialized countries due to its high morbidity and mortality rate. There are currently no curative treatments, so many investigations are studying possible therapeutic targets [1,2]. Sodium/glucose cotransporter 2 inhibitors (SGLT2i), a novel anti-diabetic drug class, have been shown to reduce the incidence of cardiovascular events and have been found to have beneficial effects even in patients without type 2 diabetes [3,4]. At present, the study of the effects of SGLT2i HF is a hot topic since the underlying mechanisms involved in the cardiac protective actions of this pharmacological treatment remain unclear. Among the proposed mechanisms of action are the shifts in myocardial metabolism from glucose consumption to ketone body utilization, reduction of oxidative stress and inhibition of the sodium-hydrogen exchanger (NHE) [5].

It has been published that SGLT2i acts within the heart to directly inhibit sodium/hydrogen exchanger 1 (NHE1) [6]. Moreover, the inhibition of cardiac NHE1 reduces cytoplasmic Na^+^ and Ca^2+^ concentrations, increasing mitochondrial Ca^2+^ levels and improving the viability of cardiomyocytes and mitochondrial function [7,8,9]. However, there are studies that question the direct inhibition of NHE1 in cardiac tissue and its effect on the regulation of intracellular Na^+^ concentration [10]. Nevertheless, the NHE family consists of many molecules involved in pH homeostasis, including the unknown molecule NHE11. Published studies about NHE11 are currently scarce. *SLC9C2* (NHE11 protein) belongs to the mammalian sperm-NHE-like subfamily (*SLC9C*). *SLC9Cs* encode an NHE-like N-terminal domain and a long non-conserved C-terminal part with similarity to the Na-transporting carboxylic acid decarboxylase transporter family [11]. Previously Wang D et al. [12] described that sperm NHE could perform as functional NHE. However, the specific activity of NHE11 is unknown. Furthermore, other sodium transporters expressed in the heart are proposed as possible targets of SGLT2i, such as the role of glucose/sodium transporters in the action mechanism of these drugs [13].

Therefore, due to the existing controversy in relation to the effect of SGLT2i on NHE and the lack of evidence on the expression and alterations in the levels of other sodium transporters in pathological and healthy human hearts, we analyzed the status of the main sodium transporters in HF. In addition, we delved into the study of the sodium/hydrogen exchangers deregulated by analyzing their protein levels and their relationship with cardiac function parameters. Furthermore, we studied the effect of empagliflozin (EMPA) on NHE11 expression in vivo, using an animal model, for the first time.

## 2. Materials and Methods

### 2.1. Human Sample Collection

In this study, we used a total of 84 human left ventricular tissue samples from patients with end-stage HF undergoing heart transplantation (mean age of 54 ± 10 years, 85% were men). Patients had previously been diagnosed with significant comorbidities, including hypertension (38%) and type 2 diabetes (34%). Patients were classified according to the functional criteria of the New York Heart Association (NYHA) and received medical treatment according to the guidelines of the European Society of Cardiology [14]. The clinical characteristics of the patients used in each study are summarized in Table 1.

A total of 16 control donors (CNT) were used (mean age 54 ± 18 years, 80% were men). The CNT samples were obtained from non-diseased hearts that could not be transplanted owing to surgical reasons or blood type incompatibility. The cause of death of these donors was a cerebrovascular event or a motor vehicle accident. All control donors had normal left ventricular function (ejection fraction > 50%) and no history of cardiac disease. Comorbidities and other echocardiographic data were not available for the CNT group in accordance with the Spanish Organic Law on Data Protection 15/1999.

The left ventricle is an integral part of the cardiovascular system; it pumps blood at a higher pressure compared with the other heart chambers, as it faces a much higher workload and mechanical afterload, so it is essential for normal function [15]. Specifically, fresh transmural samples were obtained from near the apex of the left ventricle at the time of transplantation and preserved in 0.9% NaCl at 4 °C for a maximum of 6 h from the time of removal from coronary circulation. The tissue samples were stored at –80 °C until use. A reduced time between sample receipt and storage yielded higher-quality samples, as evidenced by the RNA integrity numbers of ≥9.

This study was approved by the Ethics Committee (Biomedical Investigation Ethics Committee of La Fe University Hospital of Valencia, Valencia, Spain). Prior to tissue collection, signed informed consent was obtained from each patient. The study was conducted in accordance with the guidelines of the Declaration of Helsinki [16].

### 2.2. Transcriptomic Analysis

Transcriptome-level differences between the HF and CNT samples were investigated by means of large-scale screening of 36 heart samples (HF, n = 26; CNT, n = 10). The RNA isolation and RNA-seq procedures and analyses have been extensively described previously by Roselló-Lletí et al. [17]. Briefly, RNA extractions were performed using a PureLink™ Kit (Ambion Life Technologies, Waltham, MA, USA), and cDNA libraries were obtained following Illumina’s recommendations. Transcriptome libraries were sequenced on the SOLiD 5500 XL (Applied Biosystems, Waltham, MA, USA) platform. The data used in this publication have been deposited in the NCBI Gene Expression Omnibus (GEO) and can be retrieved using the GEO Series accession number GSE55296 (http://www.ncbi.nlm.nih.gov/geo/query/acc.cgi?acc=GSE55296, accessed on 28 April 2014).

### 2.3. NHE11 and NHE1 Protein Concentration

NHE11 and NHE1 protein levels were determined on 80 heart samples (HF, n = 70; CNT, n = 10). Protein extraction has been extensively described previously by Roselló-Lletí et al. [18]. Briefly, twenty-five milligrams of the frozen left ventricle were homogenized in an extraction buffer (2% SDS, 10 mM EDTA, 6 mM Tris–HCl, pH 7.4) in a FastPrep-24 homogenizer (MP Biomedicals) with specifically designed Lysing Matrix D tubes. The homogenates were centrifuged, and the supernatant was aliquoted. Protein concentrations of NHE11 and NHE1 were determined using a specific sandwich enzyme-linked immunosorbent assay (NHE11 ELISA Kit MBS9323174 from MyBioSource, and NHE1 ELISA Kit SEG374Hu from Cloud-Clone Corp.) following the manufacturer’s specifications. The test had a limit of detection of 0.1 and 0.052 ng/mL for NHE11 and NHE1, respectively. The intra- and inter-assay coefficients of variation were <15% for NHE11 and <12% and <10% for NHE1. No significant cross-reactivity or interference between NHE11, NHE1, and analogs was observed. The tests were quantified at 450 nm in a dual-wavelength microplate reader (Sunrise; TECAN, Tecan Ibérica Instrumentación S.L., Barcelona, Spain) using Magellan version 2.5 software (TECAN).

### 2.4. In Vivo Study

Adult male ZDF (Zucker diabetic fatty) rats (ZDF-Lepr^fa/fa^), purchased from Charles River Laboratories at 7 weeks of age with a body weight range of 200–250 g, were used in this study. The information related to their housing, feeding and treatment was extensively explained by Aragón-Herrera et al. [19]. The animals were fed ad libitum with the special rodent chow Formulab 5008 (LabDiet). The rats were accommodated in individual cages under controlled conditions. The rats were randomly divided into two groups: CNT (n = 10) with mineral drink treatment and treated (n = 12) with EMPA 30 mg/kg/d for 6 weeks. EMPA was provided by Boehringer Ingelheim Pharma GmbH&Co and administered p. o. via drinking water (dissolved by sonication) and initiated when the rats achieved fasting glucose levels of 350.75 ± 18.59 mg/dL (12 weeks old). After 6 weeks from the start of treatment, the animals were killed by decapitation. At the time of sacrifice, the rats were 19 weeks old, and the mean weight was 425 g in the EMPA-treated rats and 399 g in the untreated rats. The tissues were collected and quickly frozen on liquid nitrogen and stored at −80 °C until subsequent analysis.

*SLC9C2* (NHE11) and *SLC9A1* (NHE1) mRNA levels were determined in the left ventricle of CNT and EMPA-treated rats through RT-qPCR. RNA was extracted using a NucleoSpin kit (Macherey-Nagel GmbH & Co., Allentown, PA, USA), according to the manufacturer’s instructions. One microgram of total RNA was reverse transcribed into cDNA using the Transcriptor First Stand cDNA Synthesis Kit (F. Hoffman-La Roche Ltd., Basel, Switzerland). Perfect Master Mix SYBER^®^Green kit (with LOW ROX) and specific primers provided by Anygenes^®^ for rat *Slc9c2* (GenBank accession no. XM_008769700.2), rat *Slc9a1* (GenBank accession no. NM_012652.2), and rat *Rn18S* (GenBank accession no. NR_046237.1) were used to normalize the expression data. RT-qPCR was performed on the Stratagene MX3000p according to the manufacturer’s instructions (Agilent Technologies, Santa Clara, CA, USA). The relative expression of the *SLC9C2* and *SLC9A1* genes was calculated according to the Livak method of 2^−ΔΔCt^ [20].

The study was performed in accordance with the ARRIVE guidelines (Animals in Research: Reporting In Vivo Experiments) and the European Union Directive 2010/63. All animals were maintained and killed using protocols approved by the Animal Care Committee of the University of Santiago de Compostela in accordance with European Union Directive 2010/63. The application approval number for these experimental procedures was 15005/2015/003. The number of animals employed in the experimental procedures was the minimum necessary to develop our objectives and to ensure a pertinent statistical power.

### 2.5. Statistical Analysis

Clinical characteristics were expressed as mean ± standard deviation for continuous variables and percentages for discrete variables. Results for each variable were tested for normality using the Kolmogorov-Smirnov method. Continuous variables not following normal distribution were compared using the Mann-Whitney test, and categorical clinical variables were compared using the chi-square test. Variables with a normal distribution were compared using Student’s *t*-test for continuous variables and Fisher’s exact test for discrete variables. The Pearson and Spearman correlation coefficient was calculated to analyze the association between variables. A *p* < 0.05 was considered statistically significant. All statistical analyses were performed using SPSS software (version 20.0; IBM SPSS Inc., Armonk, NY, USA).

## 3. Results

### 3.1. Human Left Ventricle mRNA Expression of the Main Sodium Channels

**Figure 1:**
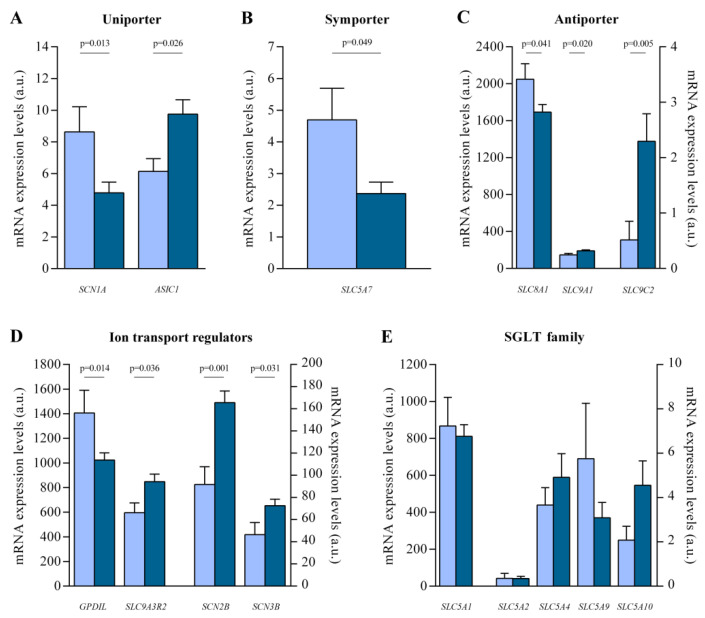
mRNA expression levels of altered genes involved in sodium transport in human heart failure (HF) hearts. (**A**) Uniporter (*SCN1A* and *ASIC1*). (**B**) Symporter (*SLC5A7*). (**C**) Antiporter (*SLC8A1*, *SCL9A1* and *SLC9C2*). (**D**) Regulators of sodium transporters (*GPD1L*, *SLC9A3R2, SCN2B* and *SCN3B*). (**E**) Sodium/glucose cotransporter (SGLT family). Bars represent mean ± SEM values. a.u., arbitrary units. Controls subjects (n = 10; light blue) and heart failure patients (n = 26; dark blue).

Differences in transcriptome-level between HF and CNT samples were investigated with a large-scale screening of 36 heart samples (HF, n = 26 and CNT, n = 10) using RNA-seq technology. We analyzed the main sodium transporters expressed in cardiac tissue (Supplementary Table S1), which were classified in relation to the type of transport used for the exchange of molecules in the cell. Among analyzed uniporter (Figure 1A), we observed *SCN1A* under-expression (FC = −1.802, *p* = 0.013), a voltage-dependent ion channel, *ASIC1* over-expression (FC = 1.588, *p* = 0.026), and a voltage-independent ion channel. Moreover, we observed differential expressions in several cotransporters (Figure 1B,C), specifically, alterations in the symporter *SLC5A7* (FC = −1.495, *p* = 0.049) and the antiporters *SLC8A1* (FC = −1.210, *p* = 0.041)*, SCL9A1* (FC = 1.170, *p* = 0.020) and *SLC9C2* (FC = 4.459, *p* = 0.005). *SLC9A1* and *SLC9C2* are sodium/hydrogen exchangers which encode the NHE1 and NHE11 proteins, respectively. Regulatory molecules of the different sodium transporters analyzed were also altered (Figure 1D), such as *GPD1L* (FC = −1.373, *p* = 0.014), *SLC9A3R2* (FC = 1.147, *p* = 0.036), *SCN2B* (FC = 1.806, *p* = 0.001), and *SCN3B* (FC = 1.564, *p* = 0.031).

Moreover, the analyzed genes that code for the different sodium/glucose transporters, potential targets of the SGLT2i, including *SLC5A1* (SGLT1 protein) and *SLC5A2* (SGLT2 protein), were detected in the human hearts of patients with HF and CNT individuals, but we did not observe statistically significant differences in the expression between both groups (Figure 1E).

### 3.2. Human Protein Expression of NHE11 and NHE1

**Figure 2:**
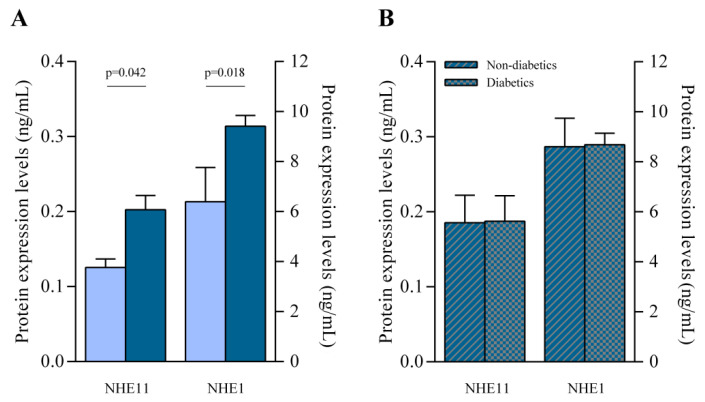
NHE11 and NHE1 protein concentration in human heart failure (HF) hearts. (**A**) NHE11 and NHE1 protein levels in control versus HF samples. (**B**) NHE11 and NHE1 protein levels in HF without type 2 diabetes versus HF with type 2 diabetes. Bars represent mean ± SEM values. Controls subjects (n = 10; light blue) and heart failure patients (n = 70; dark blue). HF without type 2 diabetes (dark blue and grey stripes), HF with type 2 diabetes (dark blue and grey squares).

In addition, using a specific enzyme-linked immunosorbent assay, with total heart samples increased to 80 (HF, n = 70 and CNT, n = 10), we found significant upregulation in the protein levels of NHE11 (FC = 1.614, *p* = 0.042) and NHE1 (FC = 1.518, *p* = 0.018) in the HF hearts (Figure 2A). Moreover, we did not find significant differences in NHE11 and NHE1 cardiac protein levels between the HF group with type 2 diabetes and those without (Figure 2B).

Furthermore, NHE11 protein expression levels showed a positive correlation with established echocardiographic parameters (Table 2), specifically left ventricular end-systolic (r = 0.334, *p* = 0.011) and left ventricular end-diastolic (r = 0.290, *p* = 0.029) diameters.

### 3.3. SLC9C2 (NHE11) and SLC9A1 (NHE1) mRNA Levels in Empagliflozin-Treated Rats

**Figure 3:**
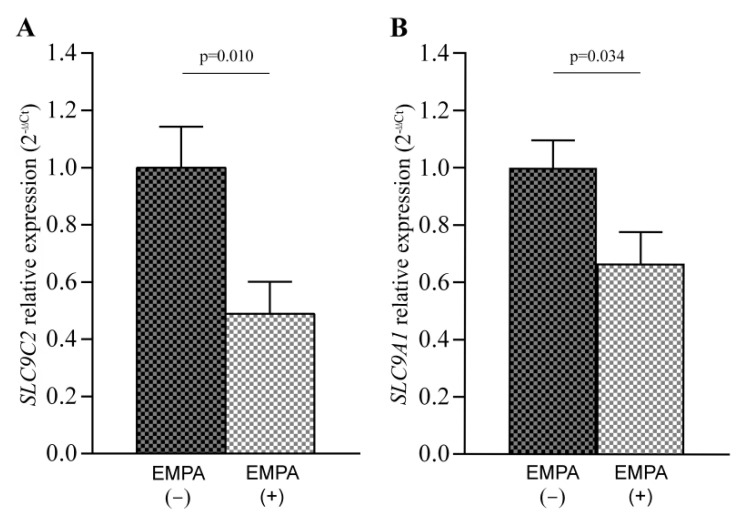
*SLC9C2* (NHE11 protein) (**A**) and *SLC9A1* (NHE1 protein) (**B**) mRNA levels in rat left ventricular tissue after treatment with empagliflozin (EMPA). Bars represent mean ± SEM values. Control rats (n = 10; black and grey squares) and rats treated with EMPA (n = 12; white and grey squares).

The effects of SGLT2i treatment on *SLC9C2* (NHE11 protein) and *SLC9A1* (NHE1 protein) mRNA levels were analyzed in the rat models’ left ventricular tissues by RT-qPCR. For this, untreated rats (n = 10) and rats treated with EMPA (n = 12) were used. Our results showed a reduction in the expression of both *SLC9C2* (FC = −2.047, *p* = 0.010; Figure 3A) and *SLC9A1* (FC = −1.504, *p* = 0.034; Figure 3B) in the rats treated with EMPA.

## 4. Discussion

Our findings showed alterations in several sodium transporters, highlighting the upregulation in two sodium/hydrogen exchangers (NHE1 and NHE11) in the left ventricular tissue of HF patients with and without diabetes. Furthermore, NHE11 protein levels were positively correlated with ventricular diameters, supporting the importance of this sodium transporter in cardiac pathology. For this reason, we analyzed, for the first time, the effect of empagliflozin, an SGLT2i, on NHE11 levels. Our results showed a relevant reduction of NHE11 mRNA levels in empagliflozin-treated rats.

Many studies have attempted to identify the pathophysiological mechanisms on which SGLT2i acts in the context of HF. One of the main proposed mechanisms is the inhibition of the sodium/hydrogen exchanger [6,7]. In this study, we demonstrated the upregulation of unknown sodium/hydrogen exchanger 11 in human heart tissue. The most relevant finding was the reduction of NHE11 mRNA expression levels in the left ventricular tissue of rats treated with empagliflozin. Currently, there is a lack of knowledge about the function of this molecule [11]; however, in the context of periodontitis, a chronic inflammatory disease, the association between the *SLC9C2* gene and systolic and diastolic blood pressure has been described [21].

NHE1 is the most studied sodium transporter in the context of cardiac pathology. In addition, it has been published that SGLT2i acts within the heart to directly inhibit NHE1 [6,7]. Controversially, Chung et al. [10] have shown that SGLT2i did not act as direct inhibitors of NHE1 activity under physiological pH conditions in an animal model. On the other hand, it has been described that SGLT2i reduces NHE1 mRNA expression in mouse cardiofibroblasts and in the left ventricle of infarcted rats, acting as an indirect inhibitor of its function [22,23]. Additionally, we confirmed the reduction of NHE1 mRNA levels in empagliflozin-treated rats. Still, little is known regarding NHE1 expression in human myocardium. In a previous study, the abundance of NHE1 protein was similar in ventricular tissue from hearts with end-stage HF and in patients with low ejection fraction [24]. We described, for the first time, the upregulation of NHE1 mRNA and protein levels in the left ventricular tissue from HF patients when compared with healthy donor hearts.

In recent years, a hypothesis that has gained strength describes that beneficial action of SGLT2i on HF is due to a systemic effect [5]. SGLT2i could act as modulators of metabolic fuel used by the myocardium, specifically reducing glucose consumption and increasing the use of ketone bodies, which ameliorate adverse left ventricle remodeling [25,26]. Previously, we observed alterations in lipids metabolism in HF patients [27], as well as in animal models treated with empagliflozin [19]. In addition, sodium/hydrogen exchangers are related to different functions in the cell. NHE1 has been related to the regulation of cellular pH, the cellular response to insulin stimuli and the process of apoptosis [28,29]. These are some of the described mechanisms on which SGLT2i acts [30,31], so it is interesting to know the modulation of NHE1 in relation to these processes. Furthermore, the structure of the isoform NHE11 is similar to NHE1 [11], but further studies are necessary to know the function of NHE11 in heart tissue.

Furthermore, the expression of sodium/glucose receptors (SGLTs) was analyzed. There is controversy regarding the presence of SGLT2 in the heart [32,33,34,35], but we have shown that SGLT2 is expressed in the human left ventricle, although at a low level. This sodium/glucose cotransporter did not seem to have a relevant role in HF since we did not find alterations in its mRNA expression. In fact, it should be noted that none of the sodium/glucose cotransporters expressed in the heart were altered in HF. However, over-expression of SGLT1 (*SLC5A1* gene) has been described in patients with ischemic cardiomyopathy and diabetic cardiomyopathy [36]. In addition, Sayour et al. [13] showed an over-expression of SGLT1 in HF patients of different etiology and the control group was composed of patients with a preserved systolic function who went through mitral valve replacement.

Our study was limited on several points, and the results must be interpreted in this context. This study does not distinguish cell types and we have determined the gene expression profiles in an animal model treated with SGLT2i, but we do not know its effect in HF patients. However, we believe that the current analyses provide substantial evidence and our findings represent a necessary first step for future research.

## 5. Conclusions

Our findings showed alterations in several sodium transports and reinforced the importance of these channels in HF progression. We described upregulation in NHE11 and NHE1 in HF patients, but only NHE11 correlated with cardiac dysfunction. In addition, the most relevant finding was the change observed in the expression of the unknown NHE11 after treatment with empagliflozin. These results propose NHE11 as a potential target of SGLT2i in cardiac tissue.

## Figures and Tables

**Table 1:** Clinical characteristics of patients with heart failure (HF).

	RNA-Seq Analysis	Protein Analysis
	HF (n = 26)	HF (n = 70)
Age (years)	53 ± 10	54 ± 10
Gender male (%)	96	84
NYHA class	III–IV	III–IV
BMI (kg/m^2^)	27 ± 5	26 ± 5
Hypercholesterolemia (%)	13	21
Prior hypertension (%)	25	38
Prior type 2 diabetes (%)	29	35
Hemoglobin (mg/mL)	14 ± 3	13 ± 2
Hematocrit (%)	40 ± 7	39 ± 6
LVEF (%)	21 ± 8	23 ± 8
LVESD (mm)	66 ± 12	60 ± 11
LVEDD (mm)	74 ± 11	68 ± 10

Data are shown as the mean value ± SD; NYHA, New York Heart Association; BMI, body mass index; LVEF, left ventricle ejection fraction; LVESD, left ventricular end-systolic diameter; LVEDD, left ventricular end-diastolic diameter.

**Table 2:** Relationships between NHEs protein levels and ventricular parameters.

	LVESD	LVEDD
NHE11	r = 0.334 *p* = 0.011	r = 0.290 *p* = 0.029
NHE1	ns	ns

LVESD, left ventricular end-systolic diameter; LVEDD, left ventricular end-diastolic diameter; ns, not significant.

## Data Availability

The data used in this publication has been deposited in the NCBI Gene Expression Omnibus (GEO) and can be retrieved using the GEO Series accession number GSE55296 (http://www.ncbi.nlm.nih.gov/geo/query/acc.cgi?acc=GSE55296, accessed on 28 April 2014).

## Supplementary Materials

The following supporting information can be downloaded at: https://www.mdpi.com/article/10.3390/pharmaceutics14101996/s1, Table S1: Genes related to sodium channels in heart failure patients.
