# Supplementary material for: Cardiac Sodium/Hydrogen Exchanger (NHE11) as a Novel Potential Target for SGLT2i in Heart Failure: A Preliminary Study
Source: Pharmaceutics. 2022 Sep 21;14(10):1996. doi: 10.3390/pharmaceutics14101996 (PMC9608584; doi:10.3390/pharmaceutics14101996)
Supplement: Supplementary file 1 [file pharmaceutics-14-01996-s001.zip › pharmaceutics-1865436-supplementary.pdf]

Supplementary Table S1. Genes related to sodium channels in heart failure patients.

	ID	GENE	PROTEIN NAME
**Voltage-dependent ion channels**			
1	ENSG00000144285	*SCN1A*	Sodium channel protein type 1 subunit alpha
2	ENSG00000136531	*SCN2A*	Sodium channel protein type 2 subunit alpha
3	ENSG00000153253	*SCN3A*	Sodium channel protein type 3 subunit alpha
4	ENSG00000007314	*SCN4A*	Sodium channel protein type 4 subunit alpha
5	ENSG00000183873	*SCN5A*	Sodium channel protein type 5 subunit alpha
6	ENSG00000136546	*SCN7A*	Sodium channel protein type 7 subunit alpha
7	ENSG00000196876	*SCN8A*	Sodium channel protein type 8 subunit alpha
8	ENSG00000169432	*SCN9A*	Sodium channel protein type 9 subunit alpha
9	ENSG00000185313	*SCN10A*	Sodium channel protein type 10 subunit alpha
10	ENSG00000168356	*SCN11A*	Sodium channel protein type 11 subunit alpha
**Voltage-independent ion channels**			
11	ENSG00000110881	*ASIC1*	Acid-sensing ion channel 1
12	ENSG00000108684	*ASIC2*	Acid-sensing ion channel 2
13	ENSG00000213199	*ASIC3*	Acid-sensing ion channel 3
14	ENSG00000111319	*SCNN1A*	Amiloride-sensitive sodium channel subunit alpha
15	ENSG00000168447	*SCNN1B*	Amiloride-sensitive sodium channel subunit beta
16	ENSG00000162572	*SCNN1D*	Amiloride-sensitive sodium channel subunit delta
17	ENSG00000166828	*SCNN1G*	Amiloride-sensitive sodium channel subunit gamma
**Symporter**			
18	ENSG00000080493	*SLC4A4*	Electrogenic sodium bicarbonate cotransporter 1
19	ENSG00000188687	*SLC4A5*	Electrogenic sodium bicarbonate cotransporter 4
20	ENSG00000033867	*SLC4A7*	Sodium bicarbonate cotransporter 3
21	ENSG00000050438	*SLC4A8*	Electroneutral sodium bicarbonate exchanger 1
22	ENSG00000100170	*SLC5A1*	Sodium/glucose cotransporter 1
23	ENSG00000140675	*SLC5A2*	Sodium/glucose cotransporter 2
24	ENSG00000198743	*SLC5A3*	Sodium/myo-inositol cotransporter
25	ENSG00000100191	*SLC5A4*	Solute carrier family 5 member 4
26	ENSG00000105641	*SLC5A5*	Sodium/iodide cotransporter
27	ENSG00000138074	*SLC5A6*	Sodium-dependent multivitamin transporter
28	ENSG00000115665	*SLC5A7*	High affinity choline transporter 1
29	ENSG00000256870	*SLC5A8*	Sodium-coupled monocarboxylate transporter 1

30	ENSG00000117834	*SLC5A9*	Sodium/glucose cotransporter 4
31	ENSG00000154025	*SLC5A10*	Sodium/glucose cotransporter 5
32	ENSG00000158865	*SLC5A11*	Sodium/myo-inositol cotransporter 2
33	ENSG00000148942	*SLC5A12*	Sodium-coupled monocarboxylate transporter 2
**Antiporter**			
34	ENSG00000163399	*ATP1A1*	Sodium/potassium-transporting ATPase subunit alpha-1
35	ENSG00000143153	*ATP1B1*	Sodium/potassium-transporting ATPase subunit beta-1
36	ENSG00000183023	*SLC8A1*	Sodium/calcium exchanger 1
37	ENSG00000118160	*SLC8A2*	Sodium/calcium exchanger 2
38	ENSG00000100678	*SLC8A3*	Sodium/calcium exchanger 3
39	ENSG00000090020	*SLC9A1*	Sodium/hydrogen exchanger 1
40	ENSG00000115616	*SLC9A2*	Sodium/hydrogen exchanger 2
41	ENSG00000066230	*SLC9A3*	Sodium/hydrogen exchanger 3
42	ENSG00000180251	*SLC9A4*	Sodium/hydrogen exchanger 4
43	ENSG00000135740	*SLC9A5*	Sodium/hydrogen exchanger 5
44	ENSG00000198689	*SLC9A6*	Sodium/hydrogen exchanger 6
45	ENSG00000065923	*SLC9A7*	Sodium/hydrogen exchanger 7
46	ENSG00000197818	*SLC9A8*	Sodium/hydrogen exchanger 8
47	ENSG00000181804	*SLC9A9*	Sodium/hydrogen exchanger 9
48	ENSG00000164037	*SLC9B1*	Sodium/hydrogen exchanger 9B1
49	ENSG00000164038	*SLC9B2*	Sodium/hydrogen exchanger 9B2
50	ENSG00000172139	*SLC9C1*	Sodium/hydrogen exchanger 10
51	ENSG00000162753	*SLC9C2*	Sodium/hydrogen exchanger 11
**Ion transport regulators**			
52	ENSG00000152642	*GPD1L*	Glycerol-3-phosphate dehydrogenase 1-like protein
53	ENSG00000172367	*PDZD3*	Na(+)/H(+) exchange regulatory cofactor NHE-RF4
54	ENSG00000105711	*SCN1B*	Sodium channel subunit beta-1
55	ENSG00000149575	*SCN2B*	Sodium channel subunit beta-2
56	ENSG00000166257	*SCN3B*	Sodium channel subunit beta-3

57	ENSG0000010906	*SLC9A3R1*	Na(+)/H(+) exchange regulatory cofactor NHE-RF1
58	ENSG00000065054	*SLC9A3R2*	Na(+)/H(+) exchange regulatory cofactor NHE-RF2

NHE-RF, Sodium/Hydrogen Exchanger Regulatory Factor.
